# The Lived Experience of Inequalities in the Provision of Treatment for Hepatitis C

**DOI:** 10.3389/fsoc.2021.649838

**Published:** 2021-06-01

**Authors:** Sarah Louise Skyrme

**Affiliations:** Policy, Ethics and Life Sciences (PEALS) Research Centre, Newcastle University, Newcastle Upon Tyne, United Kingdom

**Keywords:** stigma, healthcare provision, inequalities, risk, decision making, hepatitis C, marginalised

## Abstract

The issues of health, illness, stigma and inequalities in healthcare provision, areas that in my role as a social researcher were already of interest and concern, shifted to a different perspective when I was diagnosed with hepatitis C. From this altered position, my body and lifeworld were a nexus point for a range of ongoing challenges around staying as well as possible, and the struggle to get my healthcare needs met. There is a gap between the support provided for some ill and disabled people, and the help that they actually require. This is particularly so for conditions that are not well understood, that have a low public profile, limited funding, and/or are in some way stigmatised due to perceived differences to social norms. Hepatitis C is one such condition, it is a viral disease that is transmitted through blood-to-blood contact and it causes ongoing damage to the liver. Because of the systemic nature of the disease, individuals may struggle to cope with the demands of work and daily living, and their lifeworld and opportunities are frequently limited. It can be challenging for the patient to advocate for themselves due to low energy levels, self-blame for getting ill, and the stigma associated with the condition. The first generation of effective anti-viral drugs emerged from clinical trials in 2013, but in the United Kingdom context, access was only possible for those with advanced liver disease. Therefore, many patients felt compelled to purchase the anti-virals through Buyers Clubs, whereby generic versions of the drugs are imported for personal use at a fraction of the market cost. In this article I draw on my own lived experience of joining a Buyers Club as an example of how risks and benefits are weighed, and to explain the contexts in which decisions are shaped and made.

## Introduction

In this article I will discuss and explore my experience of becoming ill with hepatitis C and how the combination of ill health, limited treatment options, and financial constraints intersected and shaped the decision-making I engaged with. Hepatitis C is caused by a blood-borne virus that affects the liver, and transmission routes include direct blood-to-blood contact such as infected blood transfusions, needle-sharing, and unsterilized tattooing equipment (Hepatitis C Trust). Blood donations have been screened for hepatitis C since 1991 in the United Kingdom, following on from the identification of the hepatitis C virus in 1989. Due to the association with needle-sharing, and its classification as a communicable disease, individuals with hepatitis C may feel stigmatised, or be aware that it can be a stigmatising condition. Over time and if left untreated this viral infection can lead to scar tissue forming, then to cirrhosis and the associated risk of liver cancer and end-stage liver disease (Houghton, 2009). It is an RNA virus, which means that it mutates much more than a DNA virus, making it difficult for the body’s immune system to locate and then destroy (Hepatitis C Trust). There is currently no vaccine. Symptoms vary among patients and include fatigue, digestive problems, brain fog (difficulty with cognition), joint pain, jaundice, and itchy skin. Some individuals have symptoms early in the progression of the disease, while others might not have symptoms until many years have passed. Unless a specific test for the condition is done, patients who present with diverse symptoms such as stomach issues or joint pain may not get a correct diagnosis until the disease is at an advanced stage. Indeed, in a recent survey of patients with a range of liver diseases, 25% felt they “were diagnosed very late at a point when there were very few or no treatment options” (British Liver Trust, 2020, p. 1).

Until the launch of direct-acting anti-virals, the standard treatment for hepatitis C was with Interferon, usually combined with the oral drug Ribavirin. Interferon requires regular injections and takes between 6 and 12 months to potentially eradicate the virus. The treatment can cause flu-like symptoms, nausea, digestive disturbance, headaches, anxiety, fatigue, and anaemia, and has a sustained virologic response rate of 30–40%, a situation that has been deemed “woefully inadequate” (Steedman and Zobair, 2000, p. 12). From 2013 onwards, direct-acting anti-virals have been approved for treatment. They are taken in tablet form, have an elimination rate that exceeds 90% (Hurley, 2018), and, in contrast to interferon/ribavirin, are very well tolerated and can be effective in as little as 6–12 weeks. However, initial drug prices were around *£*35–40,000 in the United Kingdom and up to *$*90,000 (*£*63,000) in the United States (Hurley, 2018). These high prices resulted in healthcare providers, including the United Kingdom’s National Health Service (NHS), limiting their provision to patients with advanced liver disease. This left many patients having to deal with the ongoing and sometimes debilitating effects of the virus without access to life-changing drugs, and with the likelihood of advancing liver disease. It was in this climate of a remarkable breakthrough in drug development, which was, however, unattainable to many patients, that Buyers Clubs were set up, with patients purchasing the anti-virals at their own expense (Gornall et al., 2016).

## The Body and Lifeworld as Nexus Point

Several issues intersect in the lived experience of becoming ill with hepatitis C. My body and lifeworld, understood as my unique lived experience and perception of events (Svenaeus, 2011; Spratling, 2012), became a nexus point, a site of conflict caused not only by the virus, but by the outcomes of decisions which impacted upon me and similar patients. The experience of being on society’s margins and having a minority status (Scambler, 2009; Land and Linsk, 2013), and the interrelated experience of unequal access to support may only become apparent when individuals with hepatitis C attempt to navigate the complex setting of health and social care. This can be disorienting and is accompanied by the loss of a familiar, taken-for-granted existence (Williams and Carel, 2018), one that is replaced by a fractured terrain. As Lupton (2020) states, when serious infectious disease occurs, the usual “dimensions of everyday life can be thrown into disarray, as uncertainty, fear and anxiety are heightened” (p1). This was the case for me, I had never experienced ill health that did not improve over time. Whilst there had been a gradual increase in fatigue, joint pain and dizziness over the previous 2 years, I had only mentioned this to one doctor, who suggested it was depression and stress. Therefore, I had no frame of reference for what was happening when the symptoms intensified in the space of a few weeks, which then turned into months. I felt cut off from ordinary existence, using public transport became a dilemma, the crowds and uncertainty about getting a seat were a barrier to travel. Any type of daily activity needed an exit plan in case fatigue overwhelmed me, and my preference was to stay home if possible. My lifeworld shrank to fit this apparent “new normal”, and I felt that I had aged 20 years in a very short time. The diagnosis of hepatitis C was overwhelming, and because it is a stigmatised condition, I was cautious who I told. The combined effects of feeling ill and no longer able to cope with the physicality of daily life, and my caution in discussing this with others led to feelings of isolation and of being alone with my dilemma. Increasingly, I felt marginalised and on the edges of a social world I had previously been a part of.

A significant task in sociological work is to conduct empirical research that helps explain stigma in chronic illness and disability through indicating the causal powers of social structures like class relationships and status (Scambler, 2006). Stigma is experienced when individuals are discriminated against or have labels ascribed to them due to perceived differences to the accepted social and cultural norms of society. Where possible, individuals may avoid disclosure of their health status (Scambler, 1998), which can limit help-seeking behaviour and contribute to the low profile of conditions such as hepatitis C. Health crises can reveal discriminatory attitudes and entrenched societal inequalities (Lupton, 2020), and these shape how people cope with illness. Therefore, the physical outcomes of ill health are frequently accompanied by sociocultural, psychological, and financial struggles. The stress points touch on stigma, differential access to resources, and limited support for those living with poorly understood conditions. In my own lived experience of illness, I encountered elements of these barriers and they added to the burden of being ill. This personal experience of barriers that had previously either been invisible to me, or that I had studied from my position as a researcher, now directly impacted me and became part of daily life.

More recently, and in the aftermath of illness, an opportunity arose for me to collaborate with an animator on a funded project relating to my experience of having had hepatitis C. When completed, the short-animated film will tell the story of how illness can be accompanied by a “domino effect” where the individual’s life and their very existence become tenuous. I want the film to reach out to those facing their own health crisis, and to members of the public to help engender understanding and empathy. Indeed, it has been observed that:

… animation goes beyond just visualizing unfilmable events. It invites us to imagine, to put something of ourselves into what we see on screen, to make connections between non-realist images and reality (Roe, 2011, p. 217, p. 217).

### Animation Project

The animation is titled “In Spate” (https://sarahskyrme.uk/topic/in-spate) and it is based on key issues from my own story that have been used to develop the screenplay and the narrative running through it. We are, however, using fictionalised characters and settings in which to place the story to avoid direct representation of a sensitive topic, whilst depicting some of the challenges common to patients with hepatitis C. Prior to developing the screenplay I wrote down a linear description detailing what happened and when, to give the animator an idea of the timescale of events, such as diagnosis, becoming more ill, and how the symptoms impacted daily life. Then, during development of the animation, we discussed how to visually portray the character’s emotions, and it was through this exploration not just of what happened but how it made me feel that I recognised more fully what I had lived through. It is out of the creative and collaborative process of working on the animation project that this article has emerged. The process has motivated me to write more directly about my experiences, and to explicate how policy decisions play out at the nexus point of the individual’s life. I am using the original notes I wrote for the animator, and the further written notes and memos to myself that have arisen from my experience of using visual imagery to show emotions and events. As I have watched the development of the animation and seen representations of some of my darkest moments brought to life, albeit through a fictionalised character and setting, this visual and sequential process has helped me to find my own voice. Graphic medicine informs my conceptual approach and has been a way to explore the personal and socially relevant challenges I lived through.

## Graphic Medicine and Autoethnography

This current discussion is a form of qualitative research that uses autoethnography, a first-person narration that opens up the possibilities of sharing my insights in a “thought provoking and accessible manner” (Smith and Sparkes, 2008, p. 25). In using autoethnography I am speaking as someone who identifies as having been a patient who had hepatitis C. The process of first-person recollection that began with the animation project is helping me make sense of something that felt out of control. Graphic medicine is a source of practice and theory that is providing a methodological approach to the development of the animation through a blending of social science with an expressive form of art (Smith and Sparkes, 2008). Much of its output is based on autobiographic accounts of physical illness (Williams, 2012), mental health problems, and disability. While the primary focus is on comics and graphic novels to represent patients’ experiences, its ethos and approach are useful for exploring the disjunct between the patient’s thoughts and feelings and the provision of medical care:

Within the biomedical practice, large differences exist between how practitioners think about disease and illness and how patients experience their illness (Williams and Carel, 2018, p. 157, p. 157).

It has helped me to express a time during which the alienation caused by illness was primarily known to me as pain, fatigue and anxiety (Svenaeus, 2011), and where the gap between what I experienced and what medical care was able to offer was sometimes unbreachable. Graphic medicine blends theory and visual output to speak across and from that gap between medical knowledge and the human experience. It has been recognised as a tool for training medical students through connecting diagnosis and disease to its lived reality:

From these stories, practitioners and trainees can discover details they might not have known or fully understood about how an illness can impact a person’s daily living. Similarly, patients can learn new information from others who contend with the same illness. These new perspectives can help lessen the isolation that patients often feel … (Myers and Goldenberg, 2018, p. 158, p. 158).

Graphic medicine has become a form of therapy, theory and practice for me, it is a way to make sense of the attrition and dis-ease that accompanied my daily life and of making this legible to others. Personal accounts about the effects of chronic illness can explicate alternative stories that enrich our understanding of difference and give valuable perspectives on life and the world (Wendell, 2013). These individualised narratives can challenge the medicalised framing of illness, providing details that are missed “by the functionality of diagnostic approaches” (Priego and Farthing, 2016, p. 16). Nested within this, I am a researcher with an interest in health, illness, disability and healthcare provision, and my intent is to share the experiential dimensions of viral infection. These experiences include the loss of health, and limited support mechanisms that led to moments when I felt devoid of any agency, uncared for and alone. Those losses remain active memories that have changed how I view life now, because I experienced at first-hand how policy decisions shape the way that ill people cope and the resources they can access. Hence, from the dual positioning of being a patient and a social researcher I aim to encourage understanding and compassion through sharing my story. French (2009) contends that attempts to discover global truths about disease can eclipse the “multiplicity of local truths that are produced in the embodiment of health and illness” (p113). An empirically situated narrative is capable of describing the “local” and individualised encounter with health-related issues, it acts as a counter-narrative to the power of sanctioned stories. This positioning explicates the singular context that is not readily reflected in official accounts:

Stories are granted greater validity by the way they are secured through institutional processes and structures that give shape to everyday interactions … defining which stories are legal, which the state will support *via* welfare provision, and which will be celebrated as good or moral in culture and creative expression (Coleman-Fountain and McLaughlin, 2013, p. 143, p. 143).

This present work is also salient and timely due to the fact that hepatitis C and the issues surrounding it are infrequently spoken about, a factor that can perpetuate “stigma, marginalization and political inaction” (Harris, 2015, p. 1691).

### Living With Viral Infection

Viral infections are a source of taboos and fears based on their communicable nature. This can dehumanise patients through primarily identifying them as “vectors of disease” (French, 2009, p. 103). Narratives of contagion and blame readily conflate individuals with their disease, associating some medical problems with deviant behaviour (Scambler, 1998; Shapiro, 2008). Since the emergence of the human immunodeficiency virus (HIV) in the early 1980s, individuals with HIV have encountered negative attitudes and blaming for their health status. Particular groups such as gay men and intravenous drug users have been singled out for discrimination. Stigma can be based on fear of contagion, disease and death (Land and Linsk, 2013), and these fears are frequently generated by misinformation and the labelling of groups who are already stigmatised and discriminated against in society:

More than merely a virus, HIV serves as a vector through which flow many of society’s already-present prejudices (Hutchinson and Dhairyawan, 2018, p. 75, p. 75).

The prejudice associated with HIV is similar to that apparent in attitudes toward hepatitis C, a disease most frequently associated with drug use and needle sharing (Harris, 2015; Hurley, 2018). Individuals with this disease can also enact their own marginalisation, positioning themselves on the periphery of the social world and practicing caution regarding who they disclose their health status to. I was very aware that, although more difficult to pass on than other viruses, hepatitis C is highly infectious (NICE, 2020), a fact that causes anxiety about infecting others. As an infectious disease it is also a notifiable condition, meaning that my doctor had to report my case to the local health protection team. This measure serves to protect the public, but it can raise uncomfortable feelings of difference and isolation; public health responses tend towards exclusion and control “as opposed to inclusion and care” (French, 2009, p. 103). I knew that when crossing borders and using my passport, my health status would be flagged up to officials. It is hard not to feel a sense of shame and unease at this private information being passed on to others. There was, for me, some concern over how the information might then “leak” and be passed between agencies without my knowledge or consent. The feeling of being outside of socially accepted norms was more apparent to me at such times. I was not just a traveller, but also officially identified as a carrier of viral infection.

My anxiety about my health status being shared in unseen ways was also shaped by the misinformation that exists about the condition. Despite the work of advocacy groups, there is limited public knowledge about hepatitis C or awareness of the patient-group’s needs. This limited understanding may lead to assumptions that infected individuals are culpable and have demonstrated “a lack of personal responsibility” (Lupton, 2020, p. 5), which reinforces stereotypes:

Cultural norms of shame and blame and the labelling processes with which they are bound up never exist in a structural vacuum but invariably arise within a structural nexus (Scambler, 2009, p. 451, p. 451).

Assumptions and fears perpetuate social silencing, contributing to patients’ needs and rights being overlooked, and insufficient support provided for them at the level of policy and practice.

Public health discourse emphasizes the responsibility of the individual to stay healthy and to avoid risky and dangerous behaviours; “it is believed that one does not become ill merely out of bad luck; one becomes ill because one has courted illness” (Lupton, 1994, p. 561). The notion of the hyper-individualised (Potter et al., 2018) and digitally connected healthcare actor implies that health problems can be pre-empted; hence, for those who become ill there may be a sense of failed stewardship of the body. I felt caught up in these conflicting messages, I was an engaged actor in my health and wellbeing, but my efforts were failing, in part because of the perniciousness of hepatitis C and due to the lack of health and social support that would have eased the burden. The concept of self-management does not foreground the influence of social structures such as the economic, political and cultural shaping of society (Farmer et al., 2006). This can isolate patients, situating their predicament as a personal issue remedied through behaviour modification (Kendall et al., 2020), rather than considering how societal inequalities like inadequate provision of care and resources impinge patients’ outcomes. Nor does self-management readily acknowledge our shared vulnerabilities (Sparke and Anguelov, 2012; Potter et al., 2018) and the unpredictability of viral infection. Disease, illness and impairment can deconstruct notions of autonomous self-hood, undoing the myth of the independent self as the ideal (Coleman-Fountain and McLaughlin, 2013).

### Risk, Numbers, and Test Results

Despite limited supportive response to their needs, individuals with hepatitis C may work hard to engage with self-care, doing what they can to minimize liver damage through following healthcare advice, eating well, avoiding alcohol, and taking regular exercise. Nevertheless, the persistent, insidious nature of hepatitis C (Steedman and Zobair, 2000) requires regular monitoring of disease activity, with test results playing a significant role in many patients’ lives. In my own self-care, I tried to minimize the fatigue, joint pain and “brain fog” that were invariably present in daily life. I walked and exercised daily, abstained from alcohol and fatty foods, attended healthcare appointments and paid attention to the results of blood and liver function tests. Yet, it was apparent from my own experience how much of contemporary clinical care is exemplified through the monitoring and analysis of test results, with clinical understanding translated as diagnosis and prognosis (Shapiro, 2008). The charting of test results is undoubtedly important and necessary, but as a primary source of interactional focus between clinician and patient, this falls short of holistic care. Further to this, as a concerned patient, my interpretation of test results likely differed from the clinician’s abstracted perspective (Shapiro, 2008). For me, the numbers took on a symbolic currency as they fluctuated (Perzynski et al., 2013), leaving me alarmed when the numbers rose too high, and confused when they inexplicably dropped closer to normal, with no apparent reason for this change. Despite these undulating shifts in the data, the underlying tendency was a slide down the scale of health until my awareness of diminishing time indicated the need for action.

### Numbers as Temporality

Test results hold numerical significance because they record the status and progression of liver disease, but numbers are also significant as temporal markers, charting the passage of time. The term chronic relates to chronicity, meaning the permanence or recurrence of something, and dealing with a chronic illness results in time becoming woven into life in new ways:

The arrival of the extreme in the everyday is an interruption in “ordinary” time’s linearity because various times merge (Nayar, 2015, p. 167, p. 167).

There were occasions when time moved too rapidly, such as when my health deteriorated, yet time was also too slow, as the days, weeks and months stretched out awaiting the next appointment or test. Likewise, minutes and hours marked the time spent in waiting rooms prior to the numerous health check-ups associated with long-term care. Time was also marked out as a vigil among patients and clinicians during the months and years of waiting for updates on new treatments for hepatitis C. When news finally emerged around 2013 that successful clinical trials were resulting in first generation life-changing drugs, time was then spent waiting for the anti-virals to become available to the majority of patients.

This period of waiting was difficult for me to bear as it increased the risk that my diseased liver was going to become more damaged. When I was first diagnosed, my doctor estimated that I had 10 years until significant liver damage occurred, and as the months passed, five of those 10 years had gone, and still the clock was ticking. Now, as I write this, the 10 years have passed, and I find myself reflecting on this sobering reality and what might have happened if a cure had remained elusive. It was within this framework of temporal references that test results, news from clinical trials, and my lived experience of illness were the tangible markers of the process of this “smoldering infection” (Steedman and Zobair, 2000, p. 6). Enduring the chronicity of illness becomes part of daily life; the possibility of returning to the old normal is gradually relinquished, the hope of a new normal holds promise, but meanwhile, symptoms ebb, flow and shift under the unremitting regime of the clock face:

One way of understanding the alienating character of illness is that … the temporality of our bodies, ceases to obey our attempts to make sense of phenomena: the time of the body no longer fits into the time of the self. (Svenaeus, 2011, p. 333, p. 333)

Nayar (2015) expresses the significance of multiple temporalities, or “polychronicity”, in the illness experiences of cancer patients where:

… the sense of waiting and time passing is central to how they begin to perceive themselves, their bodies and the disease inside them (p168).

My impatience with the unpleasant elements of illness was complicated by an awareness that wishing time away, however unpleasant that time might be, appears to be absurd, because in a finite life, each moment should be savoured (Nayar, 2015). This awareness of finitude served as a powerful propellant to my actions, in the form of seeking out help and a possible solution, particularly as I was aware that one already existed and was being advocated as a reliable resource for patients. As such, it had been on the edge of my consciousness for some time, and I had read about patients on blogs and forums who had used Buyers Clubs. However, until I reached a point of realising that I had to act, it had seemed like something that other patients did, a story to follow but not personally engage with.

## Decision-Making

There were moments of stasis as exhaustion came to dominate much of my life, but also moments of inundation when the disruptive influence of pain and the associated symptoms of liver disease highlighted my body’s permeability and situational vulnerability. This was a disconcerting experience where the normality of daily life was lost:

… the unhomelikeness of illness always involves a primary alienation within the domains of our embodiment, which lends it a particularly uncanny quality … There is nowhere else to go, because the body cannot be left behind (Svenaeus, 2011, p. 341, p. 341).

Forced by circumstances, I evaluated my test results, gauged my chances of survival and considered the options for mitigating or even eliminating the liver disease. My options were constrained within a healthcare domain typified by finite resources and rising costs, where clinicians have limited time with their patients and may view their subjective experiences as secondary, unreliable, or problematic (May et al., 2006; Shapiro, 2008). Yet it was these very same subjective experiences that became the confluence point, the crux upon which my decisions were made, particularly as I believed I had reached an end-point for what healthcare could offer me, and for what I could endure. The option of using a Buyers Club to purchase my drugs was starting to coalesce into a realistic scenario for me; next I will briefly explain how a Buyers Club operates.

### Buyers Clubs

The first generation of anti-virals for the treatment of hepatitis C became available in 2013, but due to their expense the NHS was only allocating them to some patients. This was a perplexing situation whereby drug companies and pricing structures were influencing policy decisions (Sparke and Anguelov, 2012). These decisions had a significant impact on patients and became obstacles to treatment (Rosenthal and Graham, 2016) because patients who did not fit the criterion for access to the anti-virals were told to wait. This meant that patients were precluded from receiving treatment “prior to developing significant risks for ongoing liver disease” (Rosenthal and Graham, 2016, p. 6). It was in this context of life-changing drugs coming out of clinical trials, which, however, were inaccessible to many individuals, that Buyers Clubs became a source of help. These clubs had been an early means of access to cheap drugs for patients with HIV. They offer a purchasing model for legally importing anti-virals from countries such as India, where generic versions are much cheaper (Anderson, 2017). This is based on the fact that a patient can import up to 3 months of medications for personal needs. Through utilising this method, the patient can purchase the drugs via an established club who act in the role of the patient’s agent. The buyer is the patient, who is exercising their rights, and the seller is based in the country from which the medication originates, where they are operating within their laws (Anderson, 2017).

The decision I made was shaped by the knowledge that my liver disease would deteriorate and that if I left it too long, the cure might be less effective as my liver became increasingly damaged. However, the process of risk assessment and deliberation that led to my decision was accompanied by uncertainty and ambivalence (Perzynski et al., 2013) because I was acting without the direct authority of my clinician. Using a Buyers Club can create anxiety; the individual is acting as a consumer, yet public health messages encourage the avoidance of risky behaviour and adherence to healthcare advice. Therefore, to enact agency through purchasing the drugs seems counter to the behaviour expected of the “good” patient who secures the sanction of their healthcare provider prior to any medication regime. Despite these concerns I felt compelled to act, galvanised by the inescapable impact of dwindling health and strength.

### The Motivators

As discussed, paying close attention to the markers of disease progression guides patients when thinking about and making important health-related decisions. Finally, however, it was the unbearable symptoms that propelled my actions to join a Buyers Club and have the drugs imported at a fraction of their United Kingdom market cost. The symptoms were largely invisible to others and did not always fit with medicalised explanations. Ultimately, it was the combined effects of the wearing-down of illness, fear of infecting others, and a sense of existential threat that were the motivators; chronic illness brought my body to the fore, and it was no longer in the background as I went about daily life (Williams and Carel, 2018). The sensation on bad days was of energy being actively drained from my body, the exhaustion was overwhelming and lying down was the only option, eventually the exhaustion would shift back to the fatigue I had grown accustomed to. The daytime challenges were matched some nights by nightmares that left me shaken and frightened, yet none of this was discussed when medical appointments came due. My focus was on survival, on making those few minutes with the doctor count. There was little space for mention of anything beyond the virus, the associated damage to my liver, and the status of drug developments, which seemed for a long time to be out of reach.

Another factor that drove my decision was the lack of financial support available to me. The benefits system can appear punitive and bureaucratic, reliant upon medicalised explanations for conditions that are experienced by individuals in their totality, often as a form of social as well as physical dis-ease (Coleman-Fountain and McLaughlin, 2013; Sennett and Lacey, 2019). My application for financial benefits was turned down, the decision letter noted that I seemed to be coping and that steroids appeared to be helping me manage the autoimmune condition I had developed alongside the liver disease. It was difficult to comprehend the starkness of this statement, which lacked a humane recognition of need. This negative outcome acted as a motivator for the decision I took. It has been observed that methods of social support and benefits can stigmatise dependency and need as a personal failure instead of treating them as a collective responsibility (Sennett and Lacey, 2019). Hence, patients like me can feel stigmatised due to their condition and then failed by a support system that does not recognise the debilitating effects of liver disease, some of which are hidden away in the unseen liver and the hard to quantify effects of fatigue (Harris, 2015). This misrecognition, if not effacement, places an unnecessary burden on members of a patient group who are already encumbered. The expectation that I would be cared for by health and social care services and have my needs met at such a low point in my life unravelled. Not only does illness cause a rupture in the normality of life when it breaks in on us, making our “life stories chaotic” (Svenaeus, 2011, p. 339), but this is further intensified if financial and material resources are lacking. Chronic illness and the physical and material attrition which frequently accompanies it can be an incapacitating burden, and the lack of support for poorly understood conditions is further isolating, making it difficult to cope:

Coping cannot be regarded as a purely individualistic pursuit of adaptation to illness. The wider contexts of people’s lives, including their relationships with services, play an active role in enabling people to live well (Potter et al., 2018, p. 140, p. 140).

I found myself “between a rock and a hard place”, the phrase seemed so apt. I was simultaneously too ill to function in any ordinary sense of being well, and yet, apparently not ill enough to be recognised as a “rightful” beneficiary of financial support or access to anti-virals.

In the midst of this personal chaos, my choices became compromises because of the rationing of the new drugs and the limitations of social support benefits. Among the outcomes inherent in these processes is the ease with which those who are ill can fall through health and welfare gaps, making it difficult to recover and even less possible to thrive. Inequalities in the provision of medical treatments and financial support can create and entrench lacunas through who is excluded. For me, this exclusion meant that I was neither effectively treated for liver disease, nor financially supported in the absence of treatment. Restricted provision of the drugs meant that even where a specialist clinician requested access to them on behalf of a patient, as happened in my own case, limited quotas frequently prevailed:

The rationing has left many clinicians facing hard decisions and difficult conversations with patients who have seen their treatments delayed several times (Gornall et al., 2016, p. 3, p. 3).

It was in this setting of conflict, damaging delays (Gornall et al., 2016) and time spent waiting to hear if I might be granted access to the drugs *via* my clinician’s advocacy that I chose to act. The risk of doing nothing became sufficient threat that action was not only preferable, but an imperative, and I took the initial step of typing into my laptop “buy hepatitis C drugs” or some similar phrase. I then began to research and choose a reliable source and completed the necessary steps in the process of legally importing the anti-virals.

## Discussion

Drug development, healthcare policy and provision, and individual needs collide in the care and treatment of hepatitis C patients, and the same issues circulate in the wake of pandemics. Referencing the H1N1 (Swine Flu) pandemic of 2009 and the ethical concerns it raised, Sparke and Anguelov (2012) see the possibility for a global epidemiology of inequality that pays attention to the multiple mechanisms through which inequalities and affliction are connected. Understanding the biological course of disease is a vital step in building upon and improving knowledge, but without analyses of social contexts and vulnerabilities (Mastroianni, 2009), we face a “desocialization” of scientific research and inquiry (Farmer et al., 2006). Patients need access to a range of supportive services and medical care that prioritise their wellbeing, and this provision must be informed by a comprehensive understanding of needs. Placing life-saving drugs beyond the reach of patients is morally questionable. It creates unequal access to the means of eradicating viral infection and compromises the intent to improve population-level health. I lived with symptoms of liver disease that at times overwhelmed me, and yet I was classed as not ill enough to qualify for the drugs. This was a profoundly difficult situation in which my primary source of hope was the possibility that I might somehow get access to them.

The life threatening impact of viral infection highlights multifactorial issues, including the structural influences on health and medicine (Kendall et al., 2020) that shape how patients are cared for and supported. The way these intersecting matters were experienced in my life led me to recognise marginalisation and inequality not only as concepts, but as lived phenomena. For several years, the limits of health and social care were known to me as a form of physical, psychological and existential precarity that led to my decision to buy the anti-virals. My experience exemplifies pertinent concerns about who gets treatment, and how those decisions influence an individual’s life. Eradication of hepatitis C, a “pandemic infection” (Steedman and Zobair, 2000, p. 16), matters at the individual and societal level; excluding people from access to highly effective drugs without a good enough reason appears counterproductive. Yet this is what occurred in the rationing process, perhaps enabled by the fact that people with hepatitis C belong to a frequently marginalised group with a low public profile and who lack a “voice” (Gornall et al., 2016). I was fortunate, I was able to purchase the drugs and to some extent move on with my life, and this particular dilemma has largely passed due to the growing range of lower cost anti-virals now available to patients. However, that window of time and circumstance during which I found myself on the wrong side of policy decisions and the rationing of access to anti-virals illuminates persistent concerns. Inequalities, blame, risk, and stigma typify the issues and responses that emerge in the presence of viral disease, and it is within these inhibiting contexts that equitable healthcare provision is sometimes compromised. While public health messages encourage a vigilant and engaged healthcare actor who optimises activities that maintain their health, this is undermined by decisions to withhold the possibility of eradicating liver disease from patients.

## Conclusion

Viral outbreaks highlight our shared vulnerabilities and interdependencies (Sparke and Anguelov, 2012), but they also reveal biased attitudes and prejudices. Eloquent works exist that examine these factors, some of which have been briefly quoted in this article, and they have great prescience regarding the injustices wrought during health crises. Within these and similar works, recurring themes occur around the entrenching of inequalities and how decisions that result in the withholding of care and treatment from some members of society are justified. This account of my own experience offers insights into a specific time and place, when only the sickest patients (Anderson, 2017) with hepatitis C were treated. Patients like me were, in effect, told to endure further ill health until their liver disease was sufficiently advanced that they were allocated the drugs, or until the drugs became affordable. Inequalities occur in multiple ways across multiple geographies, and in this article, they intersected at the point of patients’ access to new medicines. The rationing of drugs by the NHS (Gornall et al., 2016) resulted in delays that meant patients went untreated, leaving them vulnerable to disease progression. Prolonged and increasing poor health place a strain on physical and emotional resilience, and on the material resources that individuals can draw upon. Equity in healthcare provision should be one of the basics of a fair society, optimising people’s life chances, protecting those who are vulnerable and offering an open future to the population. Health crises illuminate gaps and flaws in society, influencing the equitable flow of support, and where individuals and groups already experience ineffective provision, this is likely to be intensified.

Through explaining my own specific experience, I have sought to depict how pricing and policy decisions that are formulated and justified at the broader level actually impact at the individual level, shaping the decisions that can be made. It would be a neat ending to suggest that my quest for treatment has led to a sense of closure, which to some extent it has. What is less certain is what happens to the self after a health crisis, there is for me no sense of restitution, nor of restoration to a former life. Rather, in the wake of this, one must find a new way to live. What I experienced has been more than a disruption or detour, and some of how I am now making meaning is by using my experiences to advocate for those who have felt or do feel “vulnerable”, unwell, and/or marginalized.

## Data Availability

The original contributions presented in the study are included in the article/Supplementary Material, further inquiries can be directed to the corresponding author.
